# Impact of Obesity on Outcome for Inflammatory Bowel Disease Patients From 2008 Through 2020

**DOI:** 10.7759/cureus.70903

**Published:** 2024-10-05

**Authors:** Andrej M Sodoma, James R Pellegrini, Samuel Greenberg, Kayla West, Richard G Pellegrini, Jaspreet Singh

**Affiliations:** 1 Internal Medicine, South Shore University Hospital, Bay Shore, USA; 2 Internal Medicine, Nassau University Medical Center, East Meadow, USA; 3 Anesthesia and Critical Care, Renaissance School of Medicine, Stony Brook, USA; 4 Internal Medicine, Rocky Vista University College of Osteopathic Medicine, Englewood, USA; 5 School of Medicine, St. George's University, St. George's, GRD; 6 Gastroenterology, Northwell Health, Bay Shore, USA

**Keywords:** comorbid obesity, crohns, inflammatory bowel disease, intestinal fistula, ulcerative colıtıs

## Abstract

The incidence and prevalence of obesity have been rising in the U.S. It is known to affect the course and treatment of a variety of gastrointestinal disorders. It has been proposed to particularly worsen the symptoms of inflammatory bowel disease (IBD) and put patients at risk of treatment failure. Our study seeks to understand better the impact of obesity on morbidity, mortality, hospital length of stay (LOS), and charges in patients with IBD. The National Inpatient Sample (NIS) from 2008 to 2020 was used to identify patients over 18 diagnosed with IBD using the International Classification of Disease (ICD) 9 and 10 codes. Records were weighted per the NIS algorithm to produce accurate population estimates. Patients were classified by obesity status and then analyzed for baseline characteristics such as age, gender, race, insurance status, and various comorbidities. Various primary outcomes were analyzed. Outcomes were compared between groups, and odds ratios (ORs) were calculated using weighted logistic regression. ORs were adjusted for common co-founders. A weighted total of 2,105,418 patients admitted for IBD were included in this study. Of these, 170,475 were obese, and 1,934,943 were not obese. Obese patients were older, more complex (Charlson comorbidity index [CCI] 2.03 vs. 1.53), and more likely to be female (p<0.01) than non-obese patients. Compared to non-obese patients, IBD patients with obesity had a higher prevalence of coronary artery disease (11.35% vs. 7.87%), hyperlipidemia (29.27% vs. 15.09%), hypertension (45.38% vs. 25.60%), diabetes mellitus type 2 (27.15% vs. 9.38%), congestive heart failure (4.58% vs. 2.35%), gastroesophageal reflux disease (30.73% vs. 17.98%), and CCI (2.03% vs. 1.53%). Obese IBD patients had slightly higher odds of shock (OR 1.228, p<0.05), acute myocardial infarction (AMI, OR 1.692, p<0.01), acute kidney injury (AKI, OR 1.187, p<0.01), and chronic steroid treatment (OR 1.149, p<0.01). Conversely, obese IBD patients had lower odds of intestinal fistula (OR 0.837, CI 0.77-0.91, p<0.001). Obesity poses a diverse set of complications in IBD patients. Our study found that obese patients admitted with IBD had significantly higher odds of shock, AKI, and chronic steroid treatment. Interestingly, these patients were less likely to develop intestinal fistulas. These findings suggest further research into how obesity affects the disease course.

## Introduction

The prevalence of obesity has reached staggering levels in the United States, with an obesity prevalence of 41.9% in March of 2020 and an incidence of 7% yearly in the USA, emerging as a significant public health concern [1]. This disease has continued to trend as a major public health crisis worldwide, with prevalence rates advancing in severity across all age groups and socioeconomic classes [2]. Obesity, defined as a chronic low-grade systemic inflammation or "metabolic inflammation” with excessive accumulation of adipose tissue, has expansive and devastating impacts on the human body [3]. Beyond the association with metabolic disorders and heart disease, obesity exerts its effects on nearly all physiological systems. Obesity is associated with a myriad of metabolic abnormalities, including insulin resistance, dyslipidemia, cardiovascular disease, type 2 diabetes, and systemic inflammation [4]. Notably, the rising incidence and severity of obesity can make the incidence of these complications more common. Of particular interest is the potential exacerbation of inflammatory bowel disease (IBD) symptoms, including Crohn’s disease and ulcerative colitis, in patients with obesity.

IBD affects over 6.8 million people worldwide, with rising incidence rates observed globally over recent decades [5]. IBD represents a group of chronic inflammatory disorders of the GI tract, characterized by periods of acute inflammation followed by bouts of remission. However, the aggressive inflammatory condition often comes with debilitating symptoms such as abdominal pain, diarrhea, and fatigue. The global prevalence of IBD has been steadily increasing, with significant implications for healthcare systems and patient quality of life [6]. Concurrently, the prevalence of obesity has reached epidemic levels worldwide, posing significant challenges to public health [2]. Both IBD and obesity individually impose considerable burdens on affected individuals and healthcare systems; however, their overlap and potential congruent influence remain areas of evolving research interest.

One consistently recognized area of overlap between obesity and IBD revolves around malnutrition. Recent studies report malnutrition prevalence to be between 20% and 85% [7]. Often a result of inflammation, malnutrition is currently used as a screening tool to reliably predict clinical outcomes with various scoring systems [8]. In addition, obesity-related alterations in gut microbiota composition, adipokine secretion, and immune cell function have been proposed as potential mechanisms linking obesity to enhanced inflammatory responses and intestinal barrier dysfunction in IBD [9]. The overlap of patients with obesity and IBD requires a multifaceted analysis of patient factors of demographic, immunologic, and lifestyle that may modulate and contribute to disease susceptibility, progression, and treatment response.

Despite growing recognition of the dual burden of obesity and IBD, comprehensive investigations into their combined impact on disease pathogenesis, clinical outcomes, and therapeutic strategies remain limited. Existing studies exploring the relationship between obesity and IBD have yielded conflicting results, underscoring the need for more robust epidemiological data and studies to confirm underlying mechanisms of action [10]. Considering these implications, this retrospective cohort study aims to examine the impact of obesity on outcomes in patients with IBD. This study not only contributes to the growing body of literature on obesity and IBD but also offers insights to better understand the impact of obesity on outcomes in patients with IBD.

## Materials and methods

Data source

Our study is a retrospective cohort study using the combined 2008-2020 National Inpatient Sample (NIS), an initiative provided by the Healthcare Cost and Utilization Project (HCUP) [11]. The NIS is an all-payer database available in the United States and is maintained by the Agency for Healthcare Research and Quality (AHRQ) [11]. It comprises over 7 million unweighted records and over 35 million weighted hospital encounters annually [11]. The data provided in the database are initially unweighted, then using an algorithm provided by HCUP, they are converted to weighted data, which allows for estimates on a national level [11]. This study, like any other where NIS data is the sole data source, does not require an institutional review board (IRB) because the NIS only contains de-identified and publicly available patient information.

Study population

The NIS includes a 20% random sample of all inpatient hospitalizations from over 45 states and contains one primary diagnosis and up to 39 secondary diagnoses using the International Classification of Diseases (ICD), Tenth Revision (ICD-10), and 29 secondary diagnoses with the ICD-9-CM codes [10]. Groups were defined based on the history of IBD using ICD-9 codes (555.x, 556.x) and ICD-10 codes (K50.90, K51). Demographics were age, gender, and race (White, Black, Hispanic, and other). Also, the incidence of comorbidities, which were coronary artery disease, hyperlipidemia, hypertension, diabetes mellitus type 2, congestive heart failure, anemia, end-stage renal disease, smoking, gastroesophageal reflux disease, alcohol use disorder, liver cirrhosis, and insurance status (Medicare, Medicaid, self-pay), were compared. Again identified by ICD codes, outcomes were compared between the two groups, and odds ratios (ORs) were calculated using weighted multivariate two-stage logistic regression. Odds ratios were adjusted for common co-founders such as age, gender, race, and the Charlson comorbidity index (CCI). Patients with a new or prior diagnosis of obesity, classified as a body mass index (BMI) of more than 30, ICD-9 codes (278.0, 278.00, 278.01, 278.02), and ICD-10 codes (E66.01, E66.1, E66.3, E66.8, E66.9, Z68.30, Z68.31, Z68.32, Z68.33, Z68.34, Z68.35, Z68.36). Our primary endpoints were odds of adverse outcomes, all-cause in-hospital mortality, shock, acute myocardial infection (AMI), acute kidney injury (AKI), composite outcomes, intestinal ulcer, intestinal fistula, chronic steroid treatment, GI hemorrhage, and colorectal cancer. Secondary endpoints were the length of stay (LOS) and hospital charges.

Statistical analysis

The Pearson chi-square and Student’s t-test assessed categorical and continuous variables. The linear-by-linear association test analyzed the trends in the frequency of obesity in IBD in hospitalized patients. After adjusting for baseline characteristics and comorbidities to account for confounding variables, a two-step hierarchical multivariate regression model was used, emphasizing variables with p<0.05. These variables included age, gender, race, the CCI, and insurance payer status. Stata Version 17 by StataCorp LLC (College Station, TX) was utilized for all statistical analyses.

## Results

A weighted total of 2,105,418 patients admitted for IBD were included in this study. Of these, 170,475 were obese, and 1,934,943 were not obese. Obese IBD patients were older (50.54 years vs. 46.55 years, p < 0.0001). Obese IBD patients were more predominantly females than non-obese IBD patients (obese: 33.31% male vs. 66.69% female, non-obese: 44.29% male vs. 55.71% female, p < 0.0001). Obese patients were predominantly white (77.49% white p < 0.6480, 13.31% black p < 0.0001, 6.44% Hispanic p < 0.0167, 2.76% other p < 0.0001). Non-obese patients were also predominantly white (77.37% white p< 0.6480, 11.46% black p < 0.0001, 6.83% Hispanic p < 0.0167, 4.35% other p < 0.0001). Both obese and non-obese patients were predominantly Medicare/Medicaid patients than private insurance patients (obese: 49.8% vs. 4.2%, non-obese: 44.7% vs. 5.4%, p < 0.0001) (Table 1).

**Table 1:** Baseline characteristics of IBD patients stratified by obesity status.

Variables	Obese	Not obese	P-value
N (weighted)	170,475	1,934,943	
Age (mean in years)	50.54	46.55	<0.0001
Gender (%)			
Male	33.31	44.29	<0.0001
Female	66.69	55.71	<0.0001
Race (%)			
White	77.49	77.37	0.6480
Black	13.31	11.46	<0.0001
Hispanic	6.44	6.83	0.0167
Other	2.76	4.35	<0.0001
Comorbidities (%)			
Coronary artery disease	11.35	7.87	<0.0001
Hyperlipidemia	29.27	15.09	<0.0001
Hypertension	45.38	25.60	<0.0001
Diabetes mellitus type 2	27.15	9.38	<0.0001
Congestive heart failure	4.58	2.35	<0.0001
Anemia	9.93	11.26	<0.0001
End-stage renal disease	0.40	0.29	0.0003
Smoking	15.20	16.19	<0.0001
Alcohol use disorder	9.43	11.59	<0.0001
Liver cirrhosis	1.09	0.79	0.3934
Gastroesophageal reflux disease	30.73	17.98	<0.0001
Charlson Comorbidity Index (mean)	2.03	1.53	<0.0001
Insurance (%)			
Medicare/Medicaid	49.8001	44.7372	<0.0001
Self pay	4.2110	5.4014	<0.0001

Obese patients had a higher percentage of patients with coronary artery disease (11.35% vs 7.87%, p < 0.0001). Obese patients had a higher percentage of patients with hyperlipidemia (29.27% vs. 15.09%, p < 0.0001). Obese patients had a higher percentage of patients with hypertension (45.38% vs. 25.60%, p < 0.0001). Obese patients had a higher percentage of patients with diabetes mellitus type 2 (27.25% vs. 9.38%, p < 0.0001). Obese patients had a higher percentage of patients with congestive heart failure (4.58% vs. 2.35%, p < 0.0001). Non-obese patients had a higher percentage of patients with anemia (9.93% vs. 11.26%, p < 0.0001). Obese patients had a higher percentage of patients with end-stage renal disease (0.40% vs. 0.29%, p < 0.0001). Non-obese patients had a higher percentage of patients who smoked (15.20% vs. 16.19%, p < 0.0001). Non-obese patients had a higher percentage of patients who had been diagnosed with alcohol abuse disorder (9.43% vs. 11.59%, p < 0.0001). Obese patients had a higher percentage of patients with liver cirrhosis (1.09% vs. 0.79%, p < 0.3934). Obese patients had a higher percentage of patients with gastroesophageal reflux disease (30.73% vs. 17.98%, p < 0.0001). Obese patients were more complex, with a higher Charlson Comorbidity Index (2.03 vs. 1.53, p < 0.0001) (Table 1).

Obese IBD patients had higher odds of shock (OR 1.228, 95% CI (1.04,1.45), p < 0.0154), higher odds of AMI (OR 1.692, 95% CI (1.45,1.91), p < 0.0001), and higher odds of AKI compared to non-obese IBD patients (OR 1.187, 95% CI (1.13,1.24), p < 0.0001). Worse overall outcomes were seen in obese IBD patients than non-obese, which was performed by the summation and averaging of all-cause in-hospital morality, shock, AMI, and AKI (composite outcomes, OR 1.213, 95% CI (1.16, 1.27), p < 0.0001). No significant difference in all-cause in-hospital mortality between obese and non-obese patients was observed (OR 0.889, 95% CI (0.703,1.124), p < 0.3244). Between the two groups, these patients had relatively equal lengths of stay (LOS) (4.63 days vs. 4.61 days, OR 0.03, 95% CI (−0.02, 0.09), p < 0.2728) and hospital charges (43,840 USD vs. 38,872 USD, OR 2735, 95% CI (2156, 3314), p < 0.0001) (Table 2).

**Table 2:** Outcomes of patients admitted for inflammatory bowel disease stratified by obesity status. AMI: acute myocardial infarction; AKI: acute kidney injury; LOS: length of stay.

Outcome	Obese	Not obese	Odds ratio	95% CI	P-value
All-cause in-hospital mortality	0.2328	0.2723	0.889	0.703–1.124	0.3255
Shock	0.4628	0.3510	1.228	1.04–1.45	0.0154
AMI	1.0103	0.6254	1.692	1.45–1.91	< .0001
AKI	7.2043	5.4359	1.187	1.13–1.24	< .0001
Composite outcome	8.3940	6.3316	1.213	1.16–1.27	< .0001
Intestinal ulcer	0.91	0.96	0.984	0.87–1.11	0.7966
Intestinal fistula	2.13	2.75	0.837	0.77–0.91	< .0001
Chronic steroid treatment	7.96	6.90	1.149	1.10–1.20	< .0001
GI hemorrhage	6.75	7.01	0.965	0.92–1.01	0.1278
Colorectal cancer	0.40	0.36	0.922	0.77–1.11	0.3937
Bowel resection	6.82	6.91	0.963	0.92–1.01	0.1446
LOS (mean, days)	4.63	4.61	0.03	−0.02 to 0.09	0.2728
Charges (mean, dollars)	43840	38872	2735	2156–3314	< .0001

Obese IBD patients had higher odds of chronic steroid treatment compared to non-obese IBD patients (OR 1.149, 95% CI (1.10, 1.20), p < 0.0001). Obese IBD patients had lower odds of intestinal fistulas than non-obese IBD patients (OR 0.837, 95% CI (0.77, 0.91), p = 0.0001). No difference was found between obese and non-obese IBD patients for odds of developing or history of intestinal ulcers (OR 0.984, 95% CI (0.87, 1.11), p < 0.7966), GI hemorrhage (OR 0.837, 95% CI (0.77, 0.91), p < 0.0001), colorectal cancer (OR 0.922, 95% CI (0.77, 1.11), p < 0.3937), and bowel resection (OR 0.963, 95% CI (0.92, 1.01), p < 0.1446) (Table 2).

## Discussion

This study utilized the NIS database, identifying a total of 2,105,418 patients admitted for IBD, of whom 170,475 were classified as obese, representing 8.1% of the IBD patient population from 2008-2020, while the remaining 1,934,943 were not obese. The findings highlighted significant demographic differences between obese and non-obese IBD patients. For example, patients with obesity were statistically older and more likely to be female, highlighting demographic factors that may influence disease presentation and progression. An article by Cutolo et al. showed that sex hormones, particularly estrogen, increase humoral immunity, contributing to an increased prevalence of autoimmune diseases in females [12]. In addition, the study's comparison of CCI scores supports the notion of increased disease complexity and comorbidities, revealing that obese IBD patients had a higher CCI. Thus, patients with obesity are at a greater risk of concurrent medical conditions compared to their non-obese counterparts. This is important because the prevalence of obese IBD patients is increasing year by year, with estimates ranging from 12% to 34% of IBD patients [13-15].

Beyond demographic differences, the study revealed a statistically significant difference in the prevalence of numerous comorbidities between obese and non-obese patients with IBD. The prevalence of coronary artery disease, hyperlipidemia, hypertension, diabetes mellitus type 2, congestive heart failure, and gastroesophageal reflux disease (GERD) were significantly elevated among obese IBD patients. These findings align with previous research indicating that obesity contributes to the development and exacerbation of metabolic disorders and the severity of multiple chronic conditions [16]. The presence of these comorbidities complicates the clinical course of IBD, potentially influencing treatment strategies and patient outcomes.

The study also investigated clinical outcomes in obese versus non-obese patients with IBD, revealing both similarities and differences in disease severity and treatment response. Obese patients had slightly higher odds of experiencing adverse outcomes such as shock, AMI, AKI, and chronic steroid treatment. These findings highlight the increased vulnerability of patients with obesity and IBD to serious systemic complications and more severe disease manifestations in patients with both conditions [17]. In an article by Mahmoud and Syn, he found that obesity negatively impacted IBD patients by having worse responses to treatments with biologics and immunomodulatory [18]. The response has been linked to poor absorption, distribution, and clearance of these medications in obese patients [19].

Interestingly, obese IBD patients exhibited lower odds of developing intestinal fistulas. This finding may be due, in part, to the increased fat deposition surrounding internal organs, making it anatomically challenging to create a tract between organ structures, as found in patients with obesity. The role of mesenteric fat is multifactorial in its effects on IBD; it is a source of proinflammatory cytokines and adipokines responsible for the intense inflammation found in IBD [20,21]. However, mesenteric fat is also an active barrier to inflammation and translocated gut bacteria [22]. While this finding contrasts with the more prominent adverse outcomes observed with obesity, the results suggest a complex relationship between disease manifestation in patients with obesity and IBD.

The implications of these results extend beyond clinical management to healthcare policy and resource allocation. The findings have several important clinical implications for the management of obese patients with IBD. First, the higher prevalence of comorbidities among obese IBD patients underscores the need for comprehensive multidisciplinary care. Strategies aimed at weight management and lifestyle interventions may also be crucial in optimizing outcomes in this high-risk population [3]. Second, the increased odds of adverse clinical outcomes in obese IBD patients highlight the importance of close monitoring for disease progression and early intervention. Proactive surveillance for complications such as shock, AMI, and AKI is essential to promptly initiate appropriate therapeutic interventions and mitigate risks associated with obesity-related comorbidities [16,22].

This research has limitations. The study is a retrospective cohort study; hence, there is inherent low external validity. The data from the NIS may introduce biases related to data collection accuracy, biases of ICD coding practices among providers, readmissions of the same patient, and unmeasured confounders not included in the original articles. With NIS data, specific treatments, medications, lab values, significant clinical predictors, duration of comorbidities, duration of IBD, duration of obesity, and severity of IBD are unavailable. The only differentiation that can be made is through ICD-9/10 coding. Also, propensity scoring could not be performed with this data. Future prospective studies with detailed clinical data and longitudinal follow-up are needed to validate these findings and confirm underlying mechanisms contributing to the measured associations.

## Conclusions

Obesity poses a diverse set of complications in IBD patients. Few studies have analyzed these two disorders in conjunction. Our study found obesity in the treatment of disorders like IBD provides minimal protective effects and can increase the risk of AMI, shock, and prolonged use of steroids. Interestingly, these patients were less likely to develop intestinal fistulas. These findings are concerning and warrant stringent weight management in patients with IBD, along with further research into how obesity affects the disease course and the potential need for weight loss medications or surgical interventions in this patient population.
